# Author Correction: The hidden land use cost of upscaling cover crops

**DOI:** 10.1038/s42003-022-04188-w

**Published:** 2022-12-22

**Authors:** Bryan C. Runck, Colin K. Khoury, Patrick M. Ewing, Michael Kantar

**Affiliations:** 1 GEMS Agroinformatics Initiative, University of Minnesota, Twin Cities, St. Paul, MN 55455 USA; 2 Department of Computer Science, University of Southern California, Los Angeles, CA 90007 USA; 3 International Center for Tropical Agriculture (CIAT), Km 17, Recta Cali-Palmira, Apartado Aéreo 6713, Cali, 763537 Colombia; 4 Department of Biology, Saint Louis University, 1N. Grand Blvd., St. Louis, MO 63103 USA; 5 Department of Agronomy and Plant Genetics, University of Minnesota, St. Paul, MN 55108 USA; 6 Department of Tropical Plant and Soil Science, University of Hawaii at Manoa, Honolulu, HI 96822 USA

**Keywords:** Agriculture, Agroecology

Correction to: *Communications Biology* 10.1038/s42003-020-1022-1, published online 11 June 2020.

In the original version of the Perspective, a unit conversion error affected calculations for cereal rye, triticale, barley, and oats. Further, berseem clover yield estimates were mistranscribed from the original source. These mistakes led to errors in Supplementary Data 1, Figure 2 and in the presentation of the data in the text.

Supplementary Data 1 has now been replaced with a file containing the correct numbers.

Figure 2 has been corrected:

Original figure 2
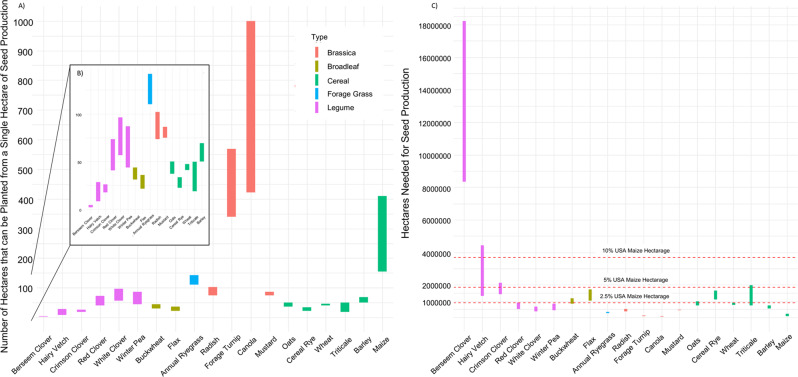


New figure 2
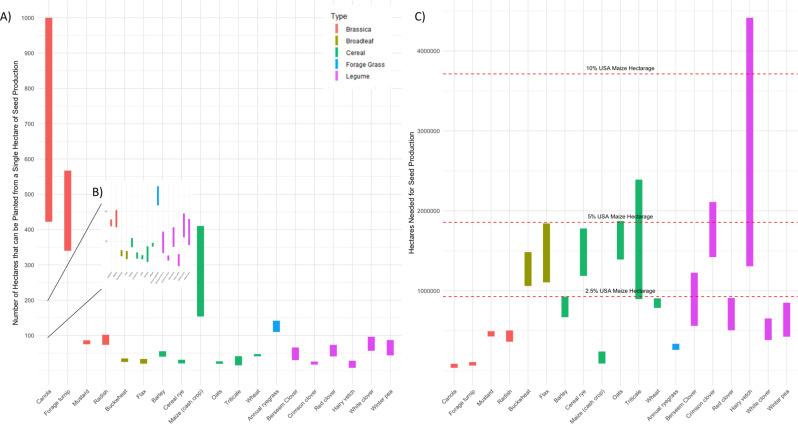


The Abstract stated: “In this Perspective, we estimate land use requirements to supply the United States maize production area with cover crop seed, finding that across 18 cover crops, on average 3.8% (median 2.0%) of current production area would be required, with the popular cover crops rye and hairy vetch requiring as much as 4.5% and 11.9%, respectively”.

The text should read: “In this Perspective, we estimate land use requirements to supply the United States maize production area with cover crop seed, finding that across 18 cover crops, on average 2.4% (median 2.1%) of current production area would be required, with the popular cover crops rye and hairy vetch requiring as much as 4.8% and 11.9%, respectively”.

In the 1^st^ paragraph of the right hand column on page 2, the text said: “(…), we find that the land requirements for production of cover crop seed would be on average 1.4 million hectares (median 746,000 ha), which is equivalent to 3.8% (median 2.0%) of the U.S. maize farmland. Rye (*Secale cereale* L.) – a midrange seed yielding cover crop and one of the most commonly used in the corn belt, would require as much as 1,661,000 hectares (4.5% of maize farmland), (…)”

The text should read: “(…) we find that the land requirements for production of cover crop seed would be on average 892,526 hectares (median 774,417 ha), which is equivalent to 2.4% (median 2.1%) of the U.S. maize farmland. Rye (*Secale cereale* L.) – a midrange seed yielding cover crop and one of the most commonly used in the corn belt, would require as much as 1,779,770 hectares (4.8% of maize farmland), (…)”

On page 3, second paragraph the text said: “Cover cropping the entire U.S. maize area would require the equivalent of as much as 18% (rye) to 49% (hairy vetch) (…)”

The text should read: “Cover cropping the entire U.S. maize area would require the equivalent of as much as 19% (rye) to 49% (hairy vetch) (…)”

This errors have now been corrected in the Perspective Article.

