# Supplementary material for: The hidden land use cost of upscaling cover crops
Source: Commun Biol. 2020 Jun 11;3:300. doi: 10.1038/s42003-020-1022-1 (PMC7289881; doi:10.1038/s42003-020-1022-1)
Supplement: Supplementary file 1 — Description of Additional Supplementary File [file 42003_2020_1022_MOESM1_ESM.pdf]

Supplementary Data 1. Yields and Seeding Rates of Commodity and Cover Crops and references for identifying cover crop seeding rates and yields which were identified from USDA statistics, extension reports, peer reviewed literature, and seed house catalogs.
